# A Comparative Study of Five Association Tests Based on CpG Set for Epigenome-Wide Association Studies

**DOI:** 10.1371/journal.pone.0156895

**Published:** 2016-06-03

**Authors:** Qiuyi Zhang, Yang Zhao, Ruyang Zhang, Yongyue Wei, Honggang Yi, Fang Shao, Feng Chen

**Affiliations:** Department of Biostatistics, School of Public Health, Nanjing Medical University, Nanjing, China, 211166; Emory University, UNITED STATES

## Abstract

An epigenome-wide association study (EWAS) is a large-scale study of human disease-associated epigenetic variation, specifically variation in DNA methylation. High throughput technologies enable simultaneous epigenetic profiling of DNA methylation at hundreds of thousands of CpGs across the genome. The clustering of correlated DNA methylation at CpGs is reportedly similar to that of linkage-disequilibrium (LD) correlation in genetic single nucleotide polymorphisms (SNP) variation. However, current analysis methods, such as the *t*-test and rank-sum test, may be underpowered to detect differentially methylated markers. We propose to test the association between the outcome (e.g case or control) and a set of CpG sites jointly. Here, we compared the performance of five CpG set analysis approaches: principal component analysis (PCA), supervised principal component analysis (SPCA), kernel principal component analysis (KPCA), sequence kernel association test (SKAT), and sliced inverse regression (SIR) with Hotelling’s *T*^*2*^ test and *t*-test using Bonferroni correction. The simulation results revealed that the first six methods can control the type I error at the significance level, while the *t*-test is conservative. SPCA and SKAT performed better than other approaches when the correlation among CpG sites was strong. For illustration, these methods were also applied to a real methylation dataset.

## Introduction

DNA polymorphisms explain only a small proportion of inheritance patterns in many complex diseases [1]. Some of the missing heritability might be explained by epigenetic variation, especially DNA methylation [2]. Indeed, the DNA methylation state, rather than DNA sequence, is more determinative of gene expression levels [3]. Further, levels of DNA methylation may “record” an individual’s environmental exposures, and thus methylation is a potential biomarker for disease diagnosis and risk stratification [4,5]. Because of the reversibility of DNA methylation, it may provide a potential therapeutic target for complex diseases, especially cancer [6,7].

Global DNA methylation status can now be profiled to determine its involvement with disease, via epigenome-wide association studies (EWASs). As an example, the HumanMethylation450 array from Illumina can assess methylation levels at more than 485,000 CpG markers [8]. The estimated proportion of DNA methylation (*β*-value) varies between 0 (unmethylated) and 1 (completely methylated). The aim of the analysis of EWAS data from case-control studies is to detect differentially methylated positions (DMPs), namely, CpGs that show a significant change in methylation between cases and controls [9]. Among the existing methods for DMP detection, the *t*-test and rank-sum test are the most commonly used [10]. Several advanced methods, such as mixture models, logistic M values, and generalized exponential tilt model, have been proposed recently [11–13].

Liu et al. have shown that the clustering of correlated DNA methylation at CpGs is similar to that of linkage disequilibrium (LD) correlation in genetic SNP variation but for a much shorter distance—the correlation is reduced by half for CpGs within 500bp, and it is weak for CpGs within 2kb [14]. Some clustering of methylated CpGs appears to be genetically driven, thus, they call these sets of correlated CpGs “GeMes”, for genetically controlled methylation clusters. Similar to LD blocks in GWASs, this type of correlated methylation structure can be a useful tool for guiding custom array design, efficient statistical approaches, and interpretation of EWASs [15].

Considering the correlation structure among CpG sites, the above methods for DMP detection, which are based on single-locus analysis, may be underpowered to detect associations. We hypothesized that an association test on a set of biologically related CpG sites may improve the power in EWAS analysis. This improvement may result from two characteristics: First, the number of tests is reduced if CpG sites are tested by set [16]. Second, a joint test can fully utilize information contained among the multiple loci.

In this study, we sought to identify joint testing methods that may offer improved power to detect associations and tested the association between disease outcome and CpG levels using several set-based methods: PCA, SPCA, KPCA, SKAT, and SIR (briefly described below). We then used simulated datasets to compare the performance of these five CpG set analysis approaches with Hotelling’s *T*^*2*^ test and *t*-test with Bonferroni correction. Additionally, we analyzed publicly available DNA methylation data from a rheumatoid arthritis (RA) dataset [17] for practical application of the methods.

## Methods

Let *i* denote the *i*th individual. For a CpG set, we used *G*_*i*1_,*G*_*i*2_,…,*G*_*ip*_ to denote DNA methylation proportions at the *p* CpG sites from the *i*th individual. When the outcome variables are dichotomous (e.g., *y* = 1/0 for case or control):

$$\text{Logit }P\left(y_{i}=1\right)=\alpha_{0}+\alpha \prime X_{i}+\beta \prime G_{i},$$

where ***X***_***i***_ = (*X*_*i1*_, *X*_*i2*_,*…*, *X*_*im*_) denotes the covariates.

### PCA

Principal component analysis (PCA) is a classical multivariate method for the analysis of non-independent variables. When the *p* explanatory variables are correlated, it is possible to use a few (*k*<<*p*) top principal components (PCs) to replace the explanatory variables in the regression analysis [18–21]. In our analysis, we used the first *k* PCs instead of *p* CpGs to test the association with the disease outcome, in which *k* is the number of PCs that explain more than 80% percent of the total variation. A *k*-df likelihood ratio test can be used to test the significance of the CpG set.

### SPCA

SPCA (supervised principal component analysis) is a supervised dimension reduction approach [22–24]. The SPCA model is:

$$\text{Logit }P\left(y_{i}=1\right)=\beta_{0}+\beta_{1}PC_{1}+\varepsilon_{j}.$$

Compared to traditional PCA, which uses all CpGs in a set to extract the PCs, only those CpGs with the strongest correlation with the outcome are used to perform SPCA, and *PC*_1_ is the first principal component. After variable selection, the test statistic $T={\overset{\wedge }{\beta }}_{1}/s.e.({\overset{\wedge }{\beta }}_{1})$ is no longer approximated well by a *t*-distribution, so we used the distribution proposed by Chen et al. for the hypothesis testing [25].

### KPCA

Kernel principal component analysis (KPCA) is a nonlinear extension of traditional PCA that has been studied intensively recently in the field of machine learning [26–29]. Given the observations, we first map the data nonlinearly into a higher-dimensional feature space *F* by

$$\begin{array}{ll} \Phi : & R^{M}\to F\\ & x\to X \end{array},$$

where ***ϕ*** is a nonlinear function. Then, a kernel matrix ***K*** is formed using the inner products of new feature vectors. A standard PCA is performed on the centralized ***K***, which is the estimate of the covariance matrix of the new feature vector in *F*_*1*_. Such a nonlinear PCA from the original data may be constructed to a linear PCA from the kernel matrix ***K***.

Commonly used kernel functions include linear kernel, polynomial kernel, radial basis function (RBF) kernel, IBS kernel, and weighted IBS kernel [30]. In particular, KPCA with linear kernel is standard linear PCA. In this study, we chose the RBF kernel due to its flexibility in choosing the associated parameter. The parameter ***σ*** is set to 0.01 and the threshold is set to 80%.

### SKAT

The sequence kernel association test (SKAT) is a supervised, flexible, computationally efficient regression method. It has been used to test for the association between a set of genetic variants and a continuous or dichotomous trait [31]. Considering the correlation among the CpG markers, we used SKAT to test the association between the trait and a set of CpGs.

To increase power, SKAT tests H_0_ by assuming each *β*_*j*_ follows an arbitrary distribution with a mean of zero and a variance of *w*_*j*_***τ***, where ***τ*** is a variance component and *w*_*j*_ is a prespecified weight for variant *j*. H_0_: ***β*** = 0 is equivalent to testing H_0_: ***τ*** = 0, which can be conveniently tested with a variance-component score test in the corresponding mixed model. The variance-component score statistic is

$$Q=(y-\overset{\wedge }{\mu })\prime_{}K(y-\overset{\wedge }{\mu }),$$

where ***K = GWWG***’, ***G*** is an *n*×*p* matrix with the (*i*, *j*)-th element being the genotype of variant *j* of subject *i*, and ***W*** = diag(*w*_*1*_, *w*_*2*_,…,*w*_*p*_) contains the weights of the *p* variants. In this study, the matrix ***G*** is quantitative and denotes the methylation values. We set *w*_*j*_ = 1; that is, all variants are weighted equally.

### SIR

Sliced inverse regression (SIR) is a novel data-analytic tool for reducing the dimension of the input variable ***G*** [32]. Instead of regressing *y* against ***G*** directly, SIR regresses ***G*** against *y* (inverse regression) by fitting ***η***(*y*) = *E*(*G*|*y*).

To perform a SIR analysis, we first standardized the explanatory variable ***G*** to $Z=\sum_{GG}^{-1/2}\left[G-E(G)\right],$ where ∑_*GG*_ is the sample covariance matrix of *G*. Second, we sliced the range of the response variable *y* into *H* intervals, *I*_*1*_,…,*I*_*h*_, and partitioned the whole dataset into several slices according to the *y* value. Let the proportion of the *y*_*i*_ that falls in the slice *h* be denoted as ${\hat{p}}_{h}=\left(1/n\right)\sum_{i=1}^{n}\delta_{h}\left(y_{i}\right)$. The value of ***δ***_*h*_(*y*_*i*_) is 0 or 1 depending on whether *y*_*i*_ falls into the *h*th slice or not. Third, we calculated the sample mean of ***Z*** within each slice, denoted as ${\hat{m}}_{h}=\left(1/n{\hat{p}}_{h}\right)\sum_{y_{i}\in I_{k}}z_{i}$. A principal component analysis was then applied to ${\hat{m}}_{h}$, extracting the most important *K*-dimensional affine subspace for tracking the inverse regression curve *E*(*G*|*y*). Finally, we output SIR after retransforming these components back to the original scale.

### Simulations

We performed simulations to evaluate the type I error and power of the five CpG set analysis approaches, in comparison to a *t*-test using Bonferroni correction and Hotelling’s *T*^*2*^ test. For the *t*-test, we extracted the minimum *P*-value as the whole *P*-value of the CpG sites (the *P* value of a CpG set). We generated the simulated datasets by using a disease model

$$\text{Logit }P(D_{i}=1)=\beta_{0}+{\sum_{j-1}^{C}\beta_{j}G}_{\text{ij}},$$

in which *C* is the number of causal CpGs. We used the program RandGen, a free program for generating random numbers, to generate the correlated CpGs [33]. Users can specify sample size, the number of variables, distributions, and correlations through the RandGen input file. If we specify the correlations between variables using the Pearson correlation parameter, then RandGen conducts a possibly time-consuming search to find the necessary copula correlation (RhoController) values to produce those desired correlations.

### Simulations based on virtual datasets

Each simulated dataset contained 1,000 cases and 1,000 controls. For each individual, we first generated methylation values using RandGen. Correlation coefficients for any pairs of CpGs were set from 0.2 to 0.8 by 0.2 increments. Here, we assumed that the CpG set contained 10 CpGs. Two scenarios were simulated; they differed by whether or not the distributions of each CpG site were the same.

**Scenario 1** (same distribution): In each situation, the mean of the corresponding distribution was 0.2/0.4/0.6 or 0.8.

**Scenario 2** (different distributions): The means of each CpG site were 0.2, 0.2, 0.3, 0.4, 0.5, 0.5, 0.6, 0.7, 0.8, and 0.8, respectively.

The outcome for each individual was determined by the above disease model. We set *C* = 0 (no causal CpG site in the set) to evaluate type I error, which was defined as the proportion of “falsely” rejected H_0_ in the 5,000 replications. To evaluate the power of the seven methods, we assumed *C* = 1 and *C* = 2. For each parameter setting, we generated 1,000 simulated datasets to calculate the power at the significance level of 0.05. Parameters of simulations are described in Table 1.

**Table 1:** Parameter settings of virtual datasets.

Simulations	Number of causal CpGs	Location of causal CpGs	Correlation coefficient (r)	Values of *β*_*j*_
Scenario 1				
1.1	0	-	0.2/0.4/0.6/0.8	-
1.2	1	1	0.2/0.4/0.6/0.8	0.5/0.6/0.7/0.8/0.9/1.0
1.3	2	1 and 2	0.2/0.4/0.6/0.8	0.1/0.2/0.3/0.4/0.5
Scenario 2				
2.1	0	-	0.2/0.4/0.6/0.8	-
2.2	1	1	0.2/0.4/0.6/0.8	0.5/0.6/0.7/0.8/0.9/1.0
2.3	1	5	0.2/0.4/0.6/0.8	0.5/0.6/0.7/0.8/0.9/1.0
2.4	1	10	0.2/0.4/0.6/0.8	0.5/0.6/0.7/0.8/0.9/1.0
2.5	2	1 and 5	0.2/0.4/0.6/0.8	0.1/0.2/0.3/0.4/0.5
2.6	2	1 and 10	0.2/0.4/0.6/0.8	0.1/0.2/0.3/0.4/0.5
2.7	2	5 and 10	0.2/0.4/0.6/0.8	0.1/0.2/0.3/0.4/0.5

### Simulations based on real DNA methylation datasets

We also simulated the CpG sets in a more realistic scenario by using a real DNA methylation dataset as the template. We used data from the Gene Expression Omnibus (GEO) generated from the Illumina HumanMethylation450 array data on whole blood (accession number GSE42861). This study examined methylation differences between RA patients (n = 354) and healthy controls (n = 335). We selected protein tyrosine phosphatase, receptor type, D (*PTPRD*) and mutL homolog 1 (*MLH1*) gene regions to generate the simulated methylation data. *PTPRD* is located on Chr 9 and *MLH1* is located on Chr 3. The CpG sites we chose are located within 1Kb of *PTPRD* and *MLH1* genes. Six CpGs on *PTPRD* (IlmnID: cg08719869, cg09371281, cg09781601, cg13723825, cg14080967, cg14458619) and nine on *MLH1* (IlmnID: cg02103401, cg04726821, cg04841293, cg05670953, cg10990993, cg11291081, cg18320188, cg21109167, cg24607398) were considered. Respectively, the correlation coefficient matrices of the two CpG sets are

$$R_{1}=\left[\begin{array}{rrrrrr} 1 & & & & & \\ 0.264 & 1 & & & & \\ 0.469 & 0.257 & 1 & & & \\ 0.778 & 0.224 & 0.458 & 1 & & \\ 0.890 & 0.248 & 0.410 & 0.819 & 1 & \\ 0.374 & 0.894 & 0.286 & 0.315 & 0.364 & 1 \end{array}\right]$$

and

$$R_{2}=\left[\begin{array}{rrrrrrrrr} 1 & & & & & & & & \\ 0.432 & 1 & & & & & & & \\ 0.138 & 0.303 & 1 & & & & & & \\ 0.331 & 0.709 & 0.292 & 1 & & & & & \\ 0.679 & 0.576 & 0.146 & 0.440 & 1 & & & & \\ 0.272 & 0.600 & 0.268 & 0.660 & 0.308 & 1 & & & \\ 0.311 & 0.421 & 0.538 & 0.518 & 0.307 & 0.443 & 1 & & \\ 0.113 & 0.585 & 0.453 & 0.668 & 0.262 & 0.417 & 0.449 & 1 & \\ 0.610 & 0.498 & 0.065 & 0.378 & 0.710 & 0.299 & 0.210 & 0.160 & 1 \end{array}\right].$$

We simulated 100 individuals with half as cases and half as controls. We set *C* = 0 to evaluate type I error and *C* = 1 to evaluate power, with 5,000 datasets produced to calculate the type I error rate, and 1,000 datasets generated for the evaluation of power. The first CpG site of each gene was defined as the causal CpG. Details of parameter settings are shown in Table 2.

**Table 2:** Parameter settings based on real methylation datasets.

Simulations	Number of causal CpGs	Location of causal CpGs	Values of *β*_*j*_
1	0	-	-
2	1	1	4.0/5.0

### Applications

The seven methods under comparison were applied to an RA DNA methylation dataset (described above). Original analysis of the dataset showed that the effect of genotype on RA risk appears to be mediated by DNA methylation changes in five genes: *GSTA2*, *PBX2*, *C6orf10*, *HLA-DQB2*, and *GPSM3* [17]. We restricted our analysis to *GSTA2* and *PBX2*, which are about 13.4 kb and 5.9 kb in length and include 6 CpGs and 51CpGs, respectively. The analyses were performed using R packages (version 3.0.2). The “superpc”, “kernlab”, “SKAT”, “dr”, and “Hotelling” packages were used to perform SPCA, KPCA, SKAT, SIR, and *T*^2^ analyses, respectively.

## Results

### Results of simulations based on virtual datasets

#### Type I error

Type I error rates of PCA, SPCA, KPCA, SKAT, SIR, *T*^2^, and *t*-test based on 10 CpGs are presented in Table 3 and Tables A-C in S1 File. Whether or not the CpGs were from the same distribution, the five set-based methods, as well as the *T*^2^ test, could control the type I error at the 0.05 significance level, while the *t*-test became increasingly conservative as the correlation among CpGs increased.

**Table 3:** Empirical Type I error rates at *α* = 0.05 level under different scenarios.

	**Same distribution (mean methylation level = 0.6)**						
***r***	**PCA**	**SPCA**	**KPCA**	**SKAT**	**SIR**	***T***^**2**^	***t*-test**
0.2	0.0504	0.0504	0.0546	0.0456	0.0492	0.0412	0.0486
0.4	0.0512	0.0506	0.0500	0.0490	0.0536	0.0498	0.0452
0.6	0.0472	0.0530	0.0572	0.0478	0.0438	0.0506	0.0340
0.8	0.0470	0.0514	0.0456	0.0446	0.0460	0.0468	0.0244
	**Different distributions**						
***r***	**PCA**	**SPCA**	**KPCA**	**SKAT**	**SIR**	***T***^**2**^	***t*-test**
0.2	0.0494	0.0496	0.0518	0.0560	0.0552	0.0544	0.0450
0.4	0.0424	0.0478	0.0482	0.0504	0.0486	0.0524	0.0422
0.6	0.0476	0.0464	0.0498	0.0510	0.0454	0.0512	0.0356
0.8	0.0420	0.0538	0.0506	0.0484	0.0482	0.0514	0.0184

#### Power

**Fig 1:**
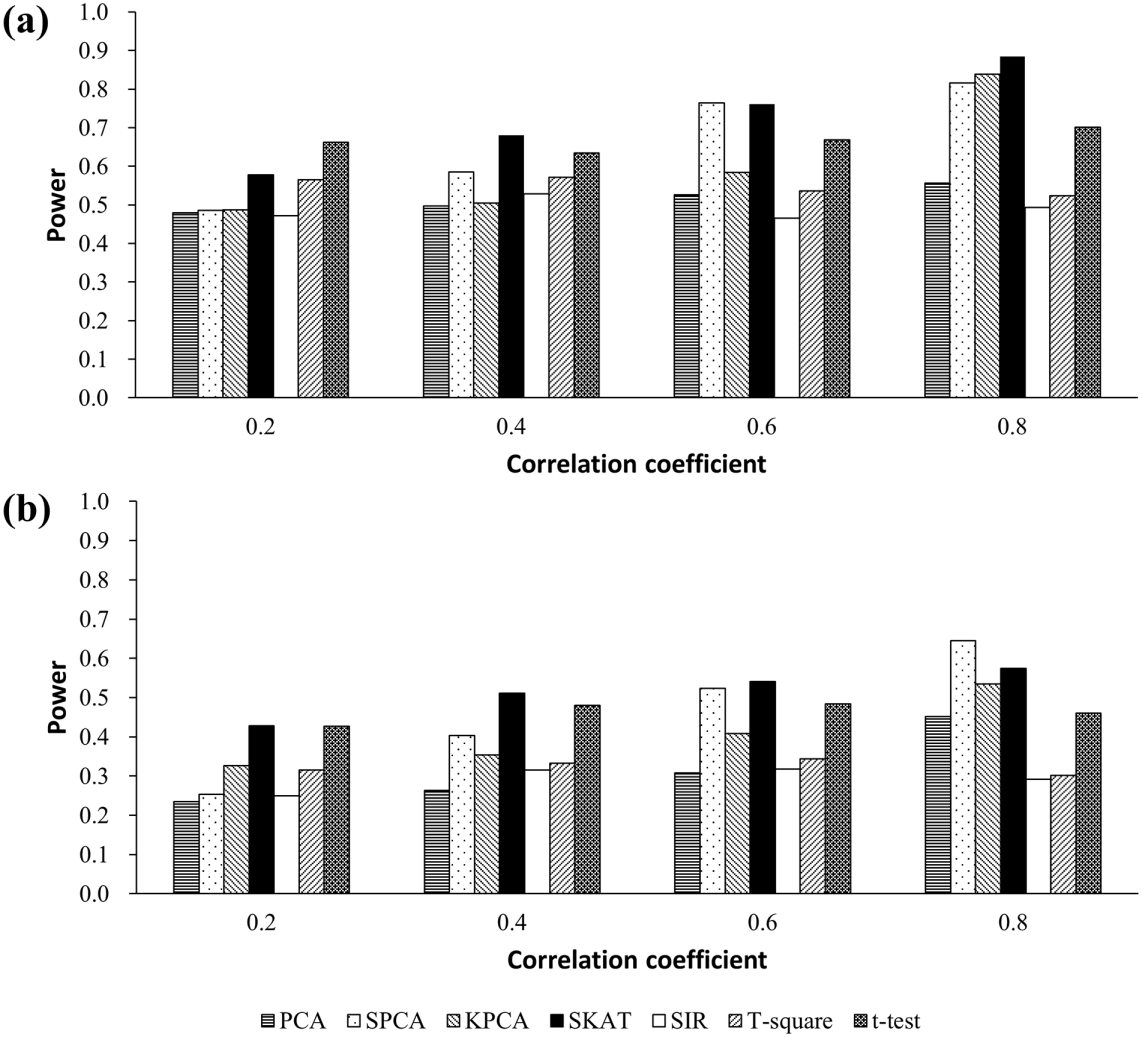
**(a) Simulated power at single causal CpG model based on 10 CpGs from the same distribution (mean methylation level = 0.6)**. The regression coefficient in the disease model, *β*_*1*_ = 0.7. **(b) Simulated power at single causal CpG model based on 10 CpGs from different distributions**. The 5^th^ CpG is set as the causal CpG. The regression coefficient in the disease model, *β*_*1*_ = 0.7.

Estimated power from scenarios 1.2 and 2.3 is presented in Fig 1. The powers of PCA, SPCA, KPCA, and SKAT increased as the correlation among CpGs increased, but there were no apparent trends for SIR, *T*^2^, and *t*-test. When correlation was strong (*r* = 0.6, *r* = 0.8), SKAT and SPCA were more powerful than the *t*-test. However, if there was weak correlation, there were no apparent differences among the three methods. We also found that the power was lower for SIR and *T*^*2*^ than for other methods, in general.

**Fig 2:**
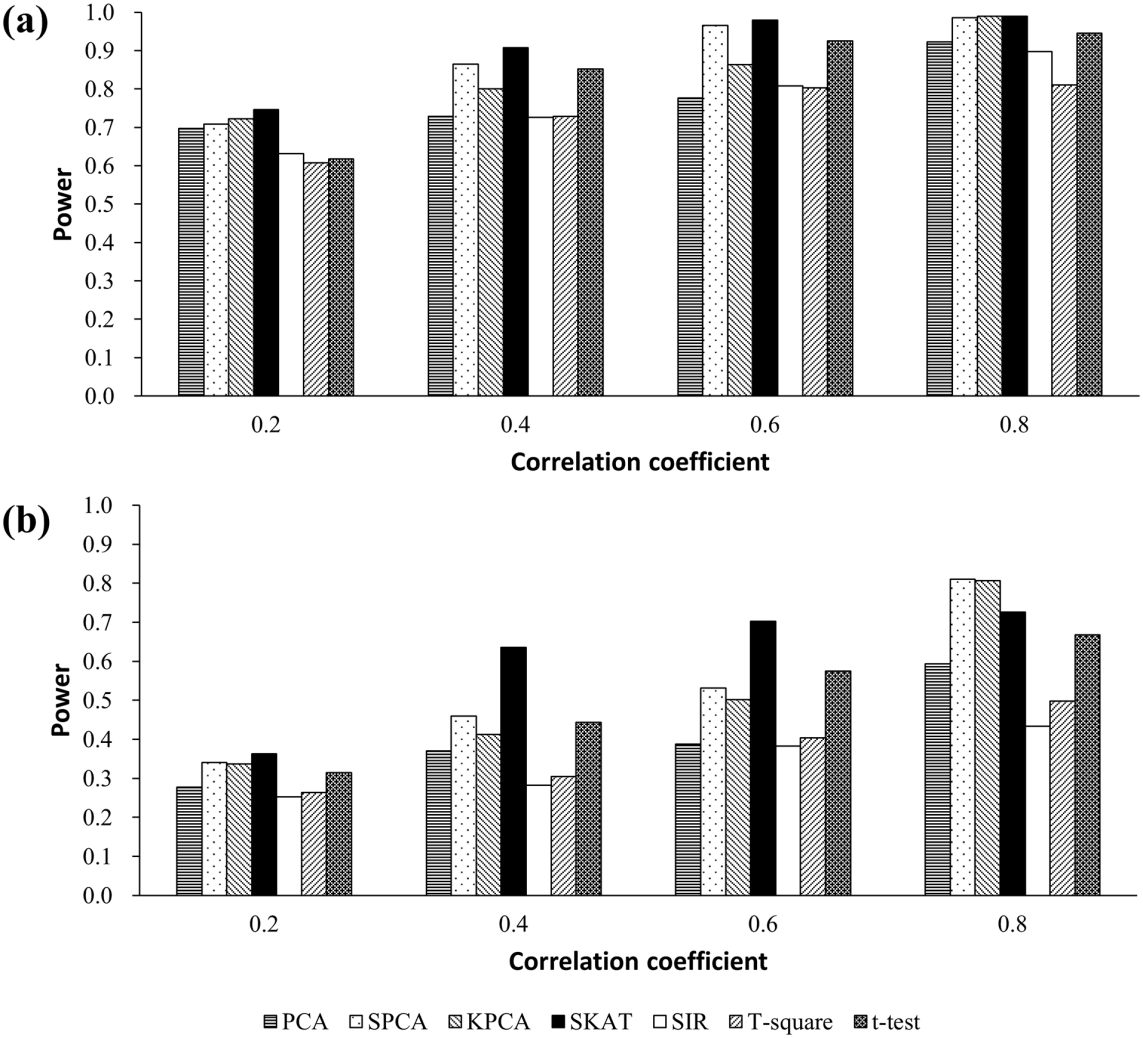
**(a) Simulated power at two causal CpGs model based on 10 CpGs from the same distribution (mean methylation level = 0.6)**. The regression coefficients in the disease model, *β*_*1*_ = *β*_*2*_ = 0.5. **(b) Simulated power at two causal CpGs model based on 10 CpGs from different distributions**. 1^st^ and 5^th^ CpGs are set as the causal CpGs. The regression coefficients in the disease model, *β*_*1*_ = *β*_*2*_ = 0.5.

Results from the simulations in scenarios 1.3 and 2.5 are presented in Fig 2. Power for all seven methods increased when the correlations became stronger. When the distributions of each CpG were the same, both SKAT and SPCA were more powerful than the other methods, independent of correlation strength. In contrast, when the distributions of each CpG were different, the powers of SKAT and SPCA were higher than *t*-test when the correlation was strong.

Considering that the average number of CpGs per gene on the HM450K array is ~17, we simulated a CpG set with 20 CpGs. Figure A in S1 File shows that SKAT and SPCA remained more powerful than other methods when the correlation was strong.

### Results of simulations based on a real DNA methylation dataset

**Fig 3:**
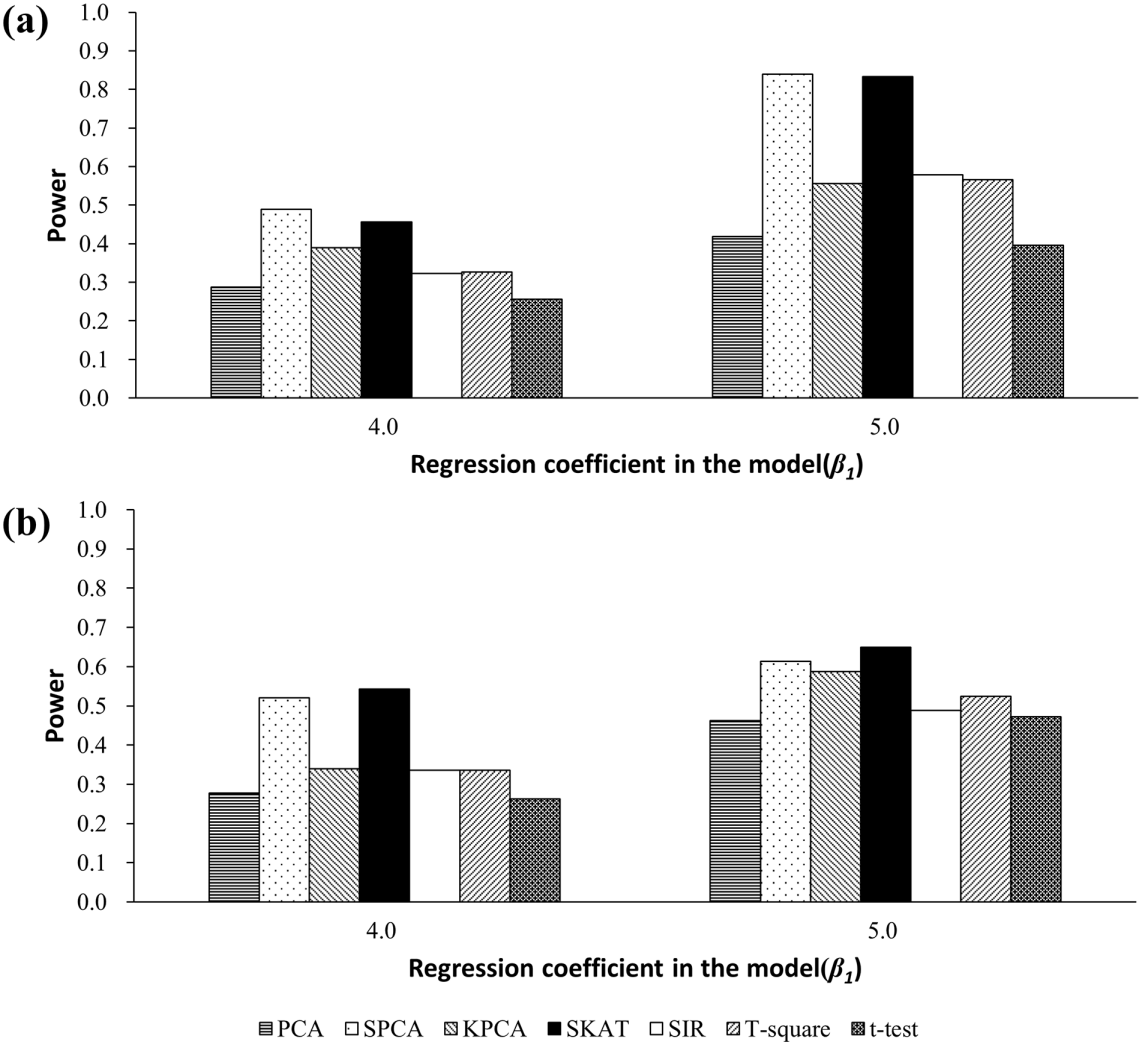
(a) Simulated power based on the *PTPRD* gene. (b) Simulated power based on the *MLH1* gene.

Table 4 presents the results based on the *PTPRD* gene. PCA, SPCA, KPCA, SKAT, SIR, and *T*^2^ could control type I error at the significance level of 0.05, while the *t*-test was conservative. Power results are presented in Fig 3(a). For both *β*_*1*_ = 4.0 and 5.0, SKAT and SPCA were more powerful than the other five methods. Among the seven methods, the power was lowest for the *t*-test. Results based on the *MLH1* gene were similar to those from the *PTPRD* gene [Table 4 and Fig 3(b)].

**Table 4:** Empirical Type I error rates based on a real methylation dataset.

Gene	PCA	SPCA	KPCA	SKAT	SIR	*T*^2^	*t*-test
*PTPRD*	0.0572	0.0578	0.0554	0.0480	0.0568	0.0584	0.0372
*MLH1*	0.0537	0.0556	0.0483	0.0514	0.0582	0.0518	0.0422

### Application to real data

We applied the seven methods to two CpG sets from an RA methylation dataset from the GEO data repository. The *P*-values for the CpG sets are presented in Table 5. For the first CpG set, the *P*-value of SPCA was 4.06E-05, the lowest of the seven methods. SKAT was second to SPCA with a *P*-value of 5.36E-04. *P*-values for PCA, *t*-test, KPCA, *T*^2^, and SIR were 1.11E-03, 1.88E-03, 2.42E-03, 3.74E-03 and 5.39E-03, respectively. For the *PBX2* gene, the result also showed that SPCA had the best performance. The *t*-test was slightly superior to SKAT. All seven approaches yielded significant results at the significance level of 0.05 and were consistent with the original report of this dataset [17].

**Table 5:** CpG set analysis results of DNA methylation datasets from epigenome studies.

Gene	Number of CpGs	*P*-value for the CpG set						
		PCA	SPCA	KPCA	SKAT	SIR	*T*^2^	*t*-test
*GSTA2*	6	1.11E-03	4.06E-05	2.42E-03	5.36E-04	5.39E-03	3.74E-03	1.88E-03
*PBX2*	51	3.32E-06	1.03E-10	9.55E-08	1.57E-08	2.80E-02	1.23E-03	4.08E-09

## Discussion

The correlation structure of the DNA methylation data enables testing of the association between the disease outcome and a set of CpGs simultaneously. Here, we demonstrate that analyzing DNA methylation data using CpG set-based analysis for epigenome-wide association studies offers superior power over individual analysis. The set-based CpG association analysis has several advantages: first, the set-based methods can “borrow” information from the correlated CpG sites; second, the set-based methods decrease the number of multiple comparisons [34–36].

In this research, we compared the performance of five CpG set analysis approaches (PCA, SPCA, KPCA, SKAT, and SIR) with Hotelling’s *T*^*2*^ test and *t*-test using Bonferroni correction. We found that all of these set-based methods can control the type I error at the target significance level. The *t*-test with Bonferroni correction is conservative, especially when the correlations between CpG sites are strong, and thus can be less powerful to identify the association between the outcome variable and CpG set. When the CpG sites in the set have high correlation with each other, SPCA and SKAT can combine their information and provide better-simulated power among the seven approaches. We suggest that SPCA and SKAT can be used for CpG set analysis and screening the association across the entire epigenome. In the RA methylation dataset, we compared the methylation differences of two CpG sets (*GSTA2* and *PBX2*) between patients and healthy controls. All these simulated studies and applications in real data analysis suggest that set-based methods may be used in DNA methylation data analysis.

SPCA is a supervised dimension reduction approach, which only includes the disease-relevant CpGs before the extraction of the principle components. Thus, this method performs better than PCA under different situations. As the KPCA does not rule out the irrelevant CpGs, the power of KPCA is inferior to SPCA but better than PCA in most occasions. We also find KPCA consumes more computational resources. SKAT uses variance component testing framework to increase test power. If the CpGs in a region are highly correlated, the reduced degree of freedom improves the statistical power. The power of SIR is almost the lowest throughout the simulations. One possible reason is that the outcome variable is binary and can be divided into only two slices. Although, in theory, Hotelling’s *T*^*2*^ test has the ability to summarize the information from correlated CpGs, it is less powerful than SPCA and PCA in our simulations. This may result from the assumptions of multiple normal distribution being violated for DNA methylation data. Thus, SIR and Hotelling’s *T*^*2*^ test are not recommended for application in CpG set analysis for EWAS studies.

In summary, we propose to use set-based method for DNA methylation data analysis and compare the performance of CpG set-based analysis for DNA methylation data. We suggest using SPCA and SKAT to improve test power. However, there remain some limitations in our study. First, the virtual simulated datasets were generated based only on multivariate beta distribution. Other distributions such as inverse logit transformation of a multivariate normal distribution should be considered. Several recent studies have noticed that measured methylation may exhibit different levels of variability in different groups, possibly due to batch effects [7]. Therefore, some new tests that capture differences in both mean and variance of methylation levels, such as semiparametric tests [13], have been proposed. We will discuss the performance of these methods in the same situation later. Second, for the methods PCA, KPCA, and SIR, further studies should be performed to identify the effect of the number of PCs on the power. Third, more complicated situations, such as the interactions between CpG sets and the methylation-mediated genetic risks in the genome-wide scan, are not covered here but will be considered in future studies.

## Supporting Information

S1 File Table A, Empirical Type I error rates at *α* = 0.05 level based on 10 CpGs from the same distribution (mean level = 0.2). Table B, Empirical Type I error rates at *α* = 0.05 level based on 10 CpGs from the same distribution (mean level = 0.4). Table C, Empirical Type I error rates at *α* = 0.05 level based on 10 CpGs from the same distribution (mean level = 0.8). Figure A, Simulated power at single causal CpG model based on 20 CpGs from the same distribution (mean methylation level = 0.6). The regression coefficient in the disease model, *β*_*1*_ = 0.7.
(DOCX)
