# Supplementary material for: A Comparative Study of Five Association Tests Based on CpG Set for Epigenome-Wide Association Studies
Source: PLoS One. 2016 Jun 3;11(6):e0156895. doi: 10.1371/journal.pone.0156895 (PMC4892473; doi:10.1371/journal.pone.0156895)
Supplement: S1 File — Table A, Empirical Type I error rates at α = 0.05 level based on 10 CpGs from the same distribution (mean level = 0.2). Table B, Empirical Type I error rates at α = 0.05 level based on 10 CpGs from the same distribution (mean level = 0.4). Table C, Empirical Type I error rates at α = 0.05 level based on 10 CpGs from the same distribution (mean level = 0.8). Figure A, Simulated power at single causal CpG model based on 20 CpGs from the same distribution (mean methylation level = 0.6). The regression coefficient in the disease model, β1 = 0.7. (DOCX) [file pone.0156895.s001.docx]

**Table A.** Empirical Type I error rates at *α*=0.05 level based on 10 CpGs from the same distribution (mean level=0.2)

| *r* | PCA | SPCA | KPCA | SKAT | SIR | *T*-square | *t*-test |
| --- | --- | --- | --- | --- | --- | --- | --- |
| 0.2 | 0.0480 | 0.0498 | 0.0544 | 0.0502 | 0.0458 | 0.0484 | 0.0402 |
| 0.4 | 0.0510 | 0.0498 | 0.0502 | 0.0492 | 0.0534 | 0.0536 | 0.0366 |
| 0.6 | 0.0552 | 0.0524 | 0.0460 | 0.0526 | 0.0554 | 0.0536 | 0.0336 |
| 0.8 | 0.0512 | 0.0508 | 0.0534 | 0.0436 | 0.0504 | 0.0494 | 0.0224 |

**Table B.** Empirical Type I error rates at *α*=0.05 level based on 10 CpGs from the same distribution (mean level=0.4)

| *r* | PCA | SPCA | KPCA | SKAT | SIR | *T*-square | *t*-test |
| --- | --- | --- | --- | --- | --- | --- | --- |
| 0.2 | 0.0508 | 0.0476 | 0.0576 | 0.0468 | 0.0438 | 0.0514 | 0.0444 |
| 0.4 | 0.0518 | 0.0476 | 0.0552 | 0.0470 | 0.0476 | 0.0520 | 0.0418 |
| 0.6 | 0.0538 | 0.0502 | 0.0494 | 0.0508 | 0.0432 | 0.0502 | 0.0330 |
| 0.8 | 0.0450 | 0.0494 | 0.0508 | 0.0490 | 0.0494 | 0.0456 | 0.0270 |

**Table C.** Empirical Type I error rates at *α*=0.05 level based on 10 CpGs from the same distribution (mean level=0.8)

| *r* | PCA | SPCA | KPCA | SKAT | SIR | T-square | t-test |
| --- | --- | --- | --- | --- | --- | --- | --- |
| 0.2 | 0.0540 | 0.0548 | 0.0510 | 0.0508 | 0.0482 | 0.0496 | 0.0510 |
| 0.4 | 0.0428 | 0.0498 | 0.0512 | 0.0472 | 0.0540 | 0.0478 | 0.0410 |
| 0.6 | 0.0506 | 0.0464 | 0.0526 | 0.0508 | 0.0502 | 0.0486 | 0.0318 |
| 0.8 | 0.0500 | 0.0458 | 0.0502 | 0.0480 | 0.0520 | 0.0440 | 0.0208 |

**Figure A.** Simulated power at single causal CpG model based on 20 CpGs from the same distribution (mean methylation level=0.6). The regression coefficient in the disease model *β_1_*=0.7.
